# Helicity‐Dependent Enzymatic Peptide Cyclization

**DOI:** 10.1002/psc.70024

**Published:** 2025-04-27

**Authors:** Canan Durukan, Jannik Faierson, Isabel van der Wal, Juan Lizandra Pérez, Sven Hennig, Tom N. Grossmann

**Affiliations:** ^1^ Department of Chemistry and Pharmaceutical Sciences VU University Amsterdam Amsterdam Netherlands; ^2^ Amsterdam Institute of Molecular and Life Sciences VU University Amsterdam Amsterdam Netherlands

**Keywords:** helix, macrocyclization, peptidomimetic, Sortase, stapled peptide

## Abstract

The secondary structure plays a crucial role in the biological activity of peptides. Various strategies have been developed to stabilize particular peptide conformations, including sequence modifications and macrocyclization approaches. Often, the interplay between conformational constraint and flexibility is central to bioactivity. Here, we investigate how peptide α‐helicity influences enzymatic head‐to‐tail cyclization using an engineered Sortase. We show that peptides with low helicity readily undergo intramolecular cyclization, while more rigid, helical peptides exhibit complex cyclization behaviors including cyclic dimer formation. These findings reveal that increased peptide rigidity can redirect enzymatic reactions from intramolecular to intermolecular processes, and demonstrates how changes in molecular rigidity can guide chemical reactivity. These insights can advance the design of peptide‐derived materials, hydrogels, and stimuli‐responsive probes.

## Graphical abstract

The ability of helical peptides to undergo head‐to‐tail cyclization reduces with increased rigidity.

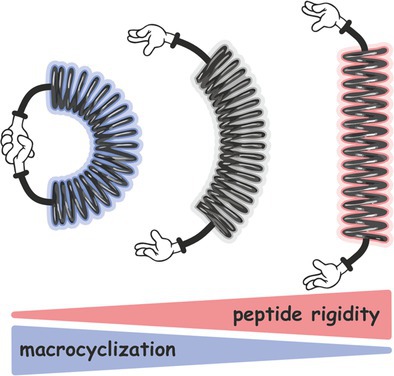

## 1 Introduction

In solution, short peptides exhibit high conformational flexibility, predominantly existing as random coil structures [1]. Upon interaction with biomolecular binding partners, however, peptide ligands often adopt well‐defined conformations [2]. The stabilization of peptide ligands in this bound‐like, bioactive confirmation has been used to enhance target affinity and bioactivity. For this purpose, various strategies have been developed including sequence modifications with proteinogenic and non‐proteinogenic amino acids, as well as macrocyclization approaches involving intra‐backbone, side‐chain to backbone, and side‐chain to side‐chain linkages [3, 4, 5]. The interplay between conformational constraint and flexibility is important when designing high‐affinity ligands [6, 7, 8].

Given the significance of α‐helices in many protein–protein interactions, helix stabilization has been a major focus of peptidomimetic approaches [9, 10]. Understanding the degree of rigidity and overall flexibility for different helix stabilization approaches is therefore of great importance. In this study, we investigated how a peptide's helix propensity influences its ability to undergo head‐to‐tail cyclization. Such macrocyclization would, in principle, require partial unfolding of the α‐helix. To investigate this aspect in more detail, we used an enzymatic peptide cyclization approach avoiding the use of activating reagents and side chain protection. Different enzymes have been reported to facilitate peptide head‐to‐tail cyclizations including transpeptidases, proteases, endopeptidases, and peptidyl ligases, as well as non‐ribosomal peptide synthetases and polyketide synthases [11, 12, 13, 14, 15, 16]. Due to its robustness and efficiency in previously reported peptide cyclizations [12, 17, 18, 19], we decided to use an engineered version of the transpeptidase Sortase A from *Staphylococcus aureus* . Herein, we describe the tuning of peptide α‐helicity using different peptidomimetic approaches and the impact of helix rigidity on the efficiency of enzyme‐mediated head‐to‐tail cyclization.

## 2 Results and Discussion

### 2.1 Engineered Sortase Performs Peptide Cyclization

**FIGURE 1:**
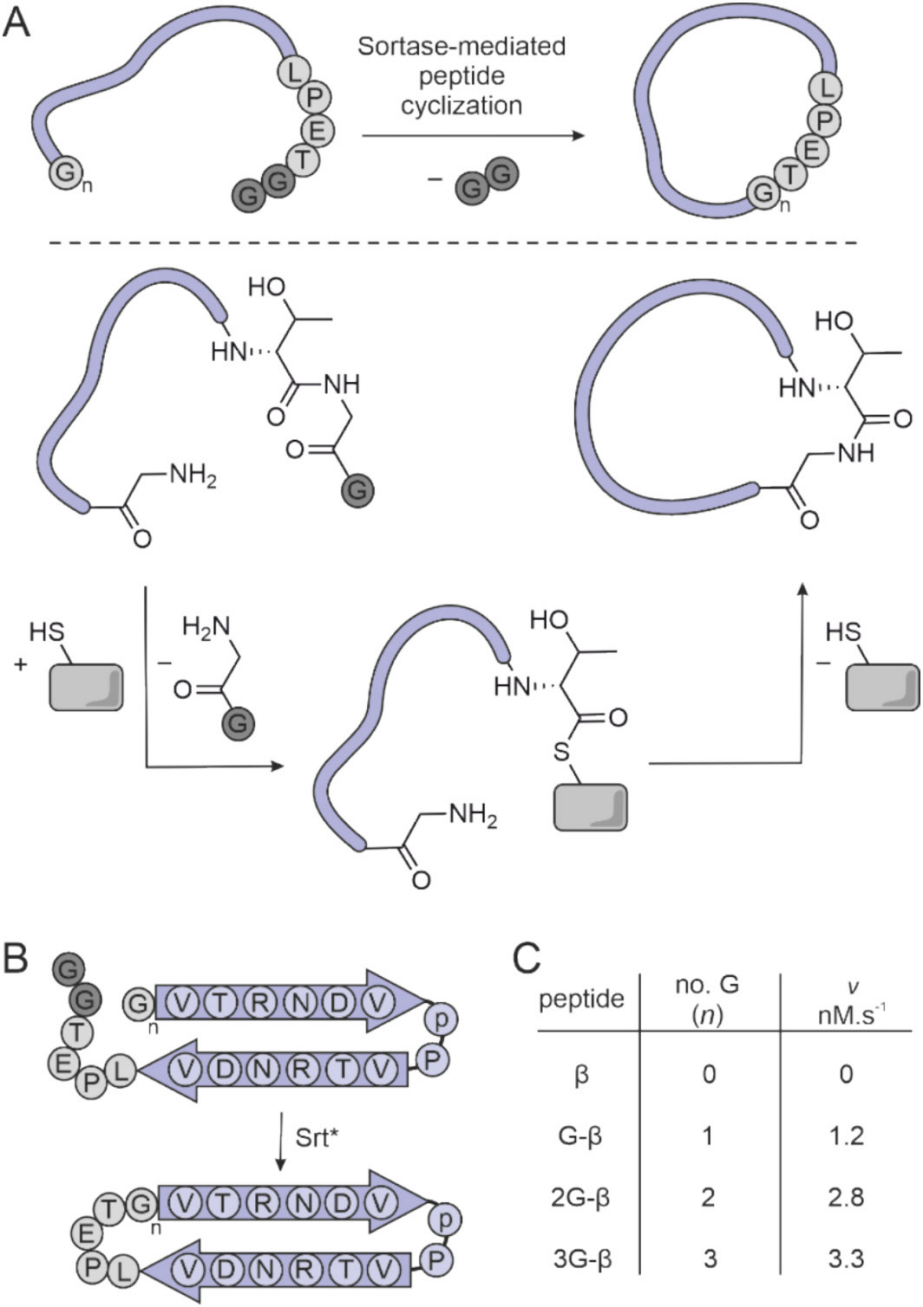
Srt*‐mediated peptide cyclization: (A) reaction scheme of Sortase‐mediated peptide cyclization. Sortase and sorting signal motifs are shown in grey, (B) cyclization of β‐sheet‐based peptides (*n* = number of N‐terminal glycine residues). (C) Initial rates (*v*) of cyclization of β‐sheet‐based peptides. For details, see Supporting Figure S3 (buffer: 20 mM HEPES, 150 mM NaCl, 5 mM CaCl_2_, 0.01% Tween‐20, 0.5 mM TCEP at pH = 7.5, peptide: *c* = 200 μM, Srt*: *c* = 60 nM).

Initially, we selected an engineered Sortase A (Srt*) with enhanced activity for cyclization reactions [20, 21, 22, 23]. Sortase‐mediated peptide cyclization is initiated by the recognition of a specific peptide sequence (LPETG, Figure 1A) known as the sorting motif. Sortase cleaves the recognition sequence between threonine and glycine (T/G) and then links the C‐terminus of the threonine to the free N‐terminal amine of a glycine. If this glycine is located terminally within the same sequence, the final acyl transfer results in the formation of a cyclic peptide (Figure 1A). It was reported that multiple N‐terminal glycine residues promote this final cyclization step [12]. To determine the optimal number of N‐terminal glycine residues, we initially used a peptide with an intrinsically cyclic structure to ensure proper cyclization (Figure 1B) [24, 25]. A panel of four peptides bearing a C‐terminal sorting motif (LPETGG) and zero to three N‐terminal glycine residues was synthesized (β–3G‐β, Supporting Figure S1, Supporting Table S1), and cyclization was attempted using Srt*. In brief, peptides (*c* = 200 μM) were incubated with Srt* (*c* = 60 nM) and the reaction progress was monitored using HPLC/MS (Supporting Figure S2). We observed the formation of the cyclization product for all peptides with at least one N‐terminal glycine (G‐β–3G‐β, Figure 1C), with the fastest reaction observed for three glycine residues (3G‐β) yielding cyclic product cy[3G‐β] (Supporting Figure S3). Notably, peptide 2G‐β bearing two glycine residues also showed good conversion (ca. 85% activity relative to 3G‐β, Figure 1C).

### 2.2 Design and Synthesis of Helical Peptides

**FIGURE 2:**
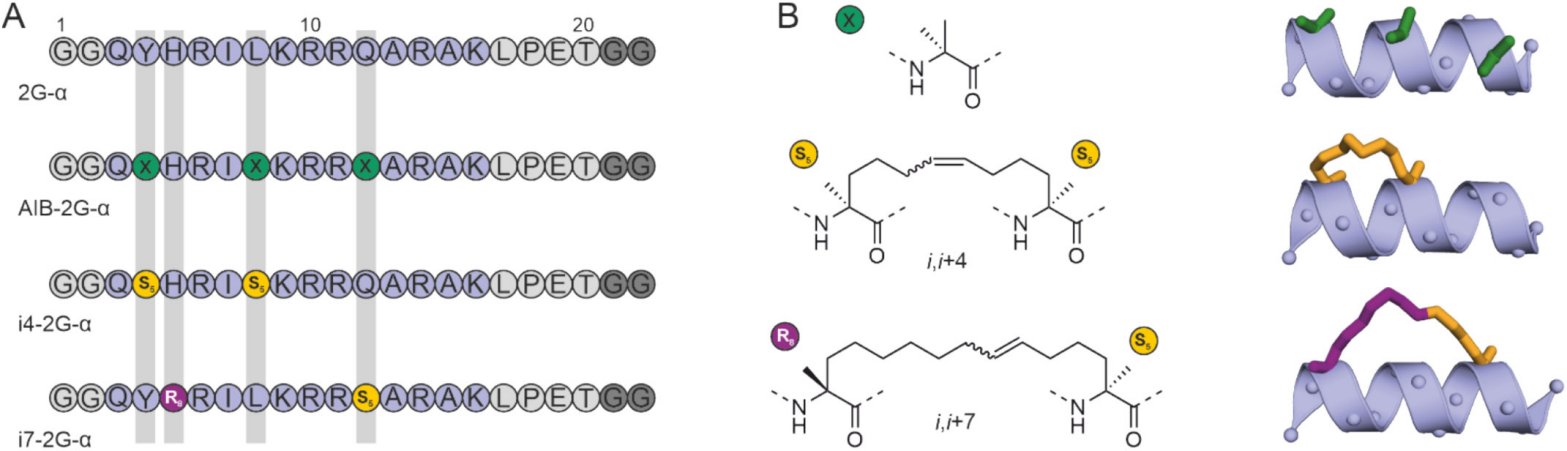
Design of helical peptides: (A) peptide sequences indicating the position of non‐proteinogenic amino acids (x: α‐aminoisobutyric acid (AIB), *S*_5_: *S*‐2‐(4′‐pentenyl)alanine, *R*_8_: *R*‐2‐(7′‐octenyl)alanine). (B) Left: chemical structures of non‐proteinogenic building blocks found in peptides AIB‐2G‐α, i4‐2G‐α, and i7‐2G‐α, respectively. Right: models of the building blocks within an α‐helix (peptide in cartoon representation).

Having confirmed the general feasibility of peptide cyclization using Sortase, we aimed to design a series of peptides with varying degrees of α‐helicity. As a starting point, we selected a peptide originating from a helical segment of the transcription factor protein NF‐YA [26, 27, 28]. The NF‐YA‐derived sequence (Figure 2A, top blue) was equipped with two N‐terminal glycine residues and the C‐terminal LPETGG motif to provide peptide 2G‐α. In addition, three peptide sequences with helix‐promoting modifications were designed (Figure 2B). This included the introduction of three α‐aminoisobutyric acid (AIB) building blocks to provide peptide AIB‐2G‐α [29, 30, 31, 32, 33, 34, 35]. AIB is an achiral derivative of alanine featuring a second α‐carbon methyl group which promotes the helical conformation of peptides [36]. In addition, two hydrocarbon stapled peptides were designed that feature an *i*,*i* + 4 and *i*,*i* + 7 side chain‐to‐side chain crosslink, respectively, providing peptide i4‐2G‐α and i7‐2G‐α (Figure 2B). In hydrocarbon peptide stapling, α‐methyl and α‐alkenyl amino acids are introduced during solid‐phase peptide synthesis which are then crosslinked using ring‐closing olefin metathesis [25, 37, 38, 39, 40]. For the *i*,*i* + 4 staple bridging one helical turn in i4‐2G‐α, two *S*‐2‐(4′‐pentenyl)alanines (*S*_5_) were incorporated. In i7‐2G‐α, *R*‐2‐(7′‐octenyl)alanine (*R*_8_) was incorporated at position *i* and *S*_5_ at position *i* + 7 to provide a staple bridging two helical turns. All four peptides were obtained using solid‐phase synthesis and processed through established macrocyclization and purification protocols (Supporting Figure S4) [41].

### 2.3 Characterization of Helical Peptides

**FIGURE 3:**
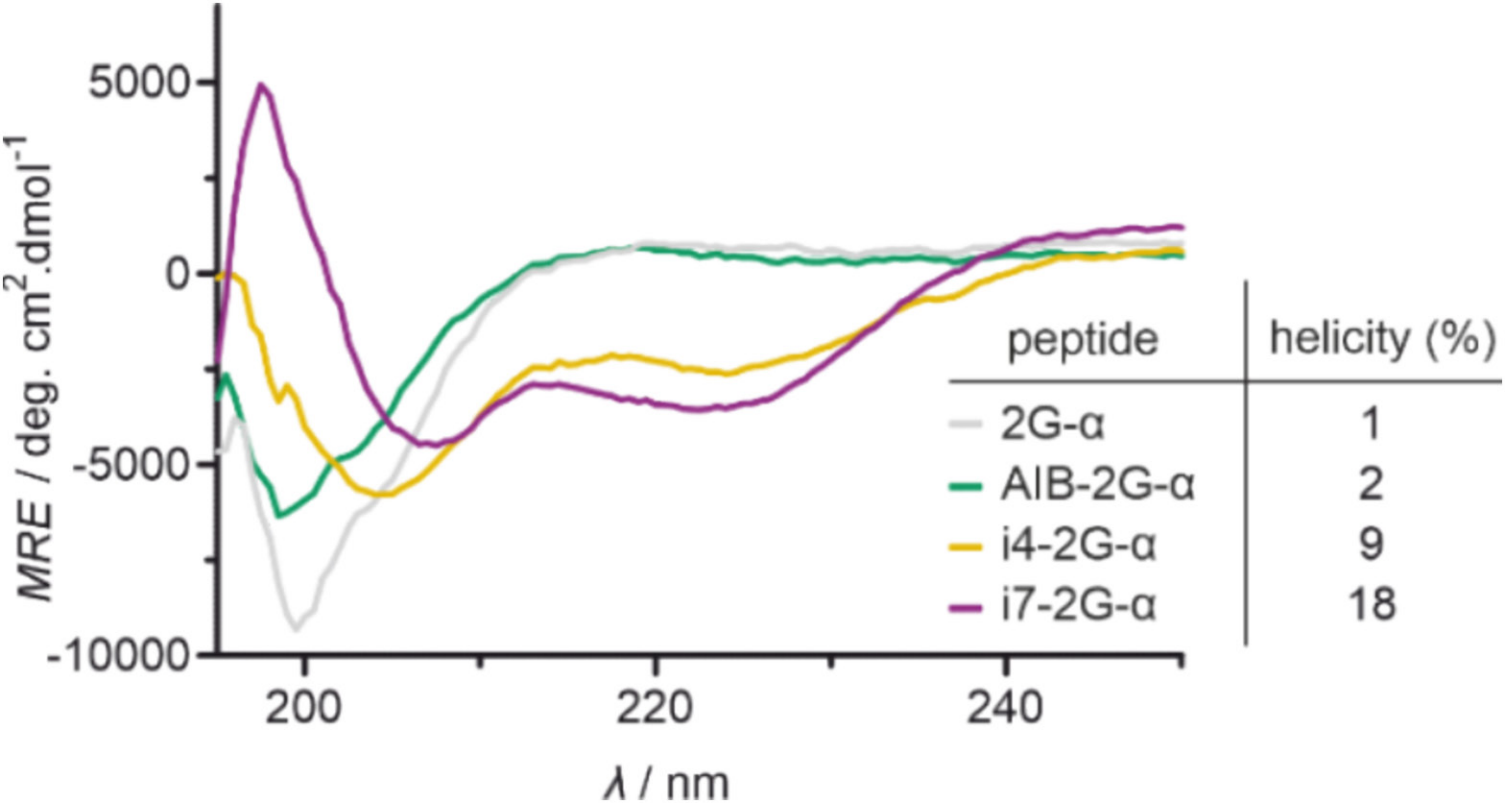
Circular dichroism (CD) spectra of peptides 2G‐α, AIB‐2G‐α, i4‐2G‐α, i7‐2G‐α (*c* = 10 μM in 10 mM phosphate, pH 7.4). Helicities were determined using the BeStSel webserver [42, 43, 44, 45, 46].

To assess the α‐helicity of these peptides, circular dichroism (CD) spectroscopy was performed. CD spectra were measured in sodium phosphate buffer at pH 7.4. The spectra of 2G‐α and AIB‐2G‐α lacked the characteristic helix signal at *λ* = 222 nm (Figure 3). Based on the Beta Structure Selection (BeStSEL) tool, a Web server for CD‐based secondary structure analysis, both peptides exhibited very low helical content (1% and 2% helicity, respectively) [42, 43]. Notably, both stapled peptides displayed considerably increased helicity, as demonstrated by the characteristic double minima in their CD spectra at approximately 208 and 222 nm (Figure 3). Among these, *i*,*i* + 7 stapled peptide i7‐2G‐α demonstrated a higher helical content than i4‐2G‐α bearing an *i*,*i* + 4 staple (18% vs. 9% helicity). Peptide 2G‐α's low helicity is consistent with the lack of helix‐promoting modifications. Interestingly, AIB‐2G‐α also showed very low helicity, indicating that α‐methylation via AIB substitution did not significantly impact helicity in this context. Notably, in peptide i7‐2G‐α, the *i*,*i* + 7 crosslink spanning two turns of the α‐helix appears to have a stronger helix‐inducing effect than the one‐turn‐spanning *i*,*i* + 4 crosslink in i4‐2G‐α.

### 2.4 Cyclization Efficiency Depends on Helicity

**FIGURE 4:**
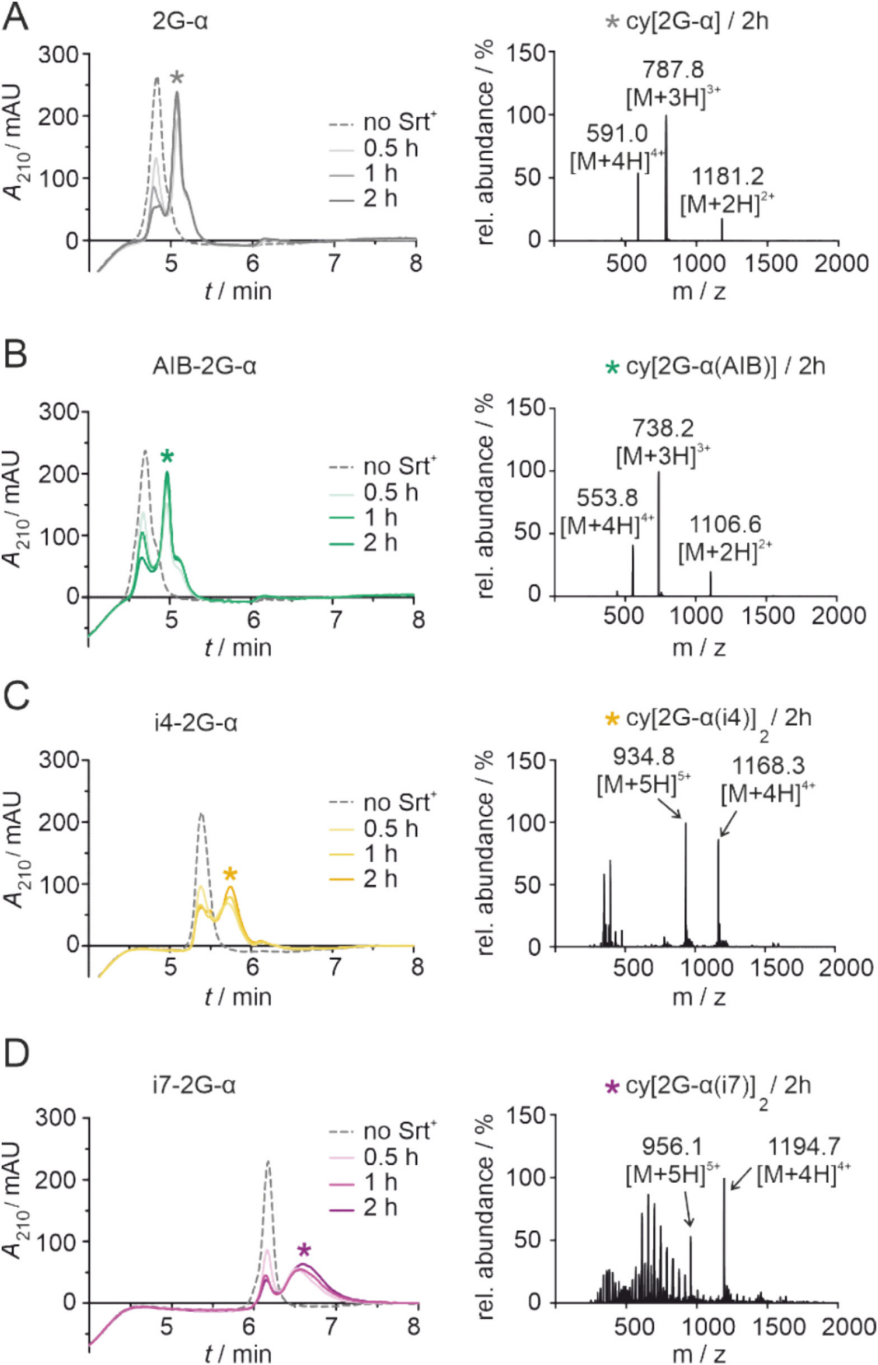
HPLC‐MS analysis of cyclization reactions of peptide 2G‐α (A), AIB‐2G‐α (B), i4‐2G‐α (C), i7‐2G‐α (D) using Srt^+^ (peptide: *c* = 100 μM, Srt^+^: *c* = 1 μM, buffer: 20 mM HEPES, pH 7.5, 150 mM NaCl, 5 mM CaCl_2_, 0.01% Tween‐20, 0.5 mM TCEP). Left: HPLC chromatograms showing traces before enzyme addition (dashed line) and after 0.5, 1, and 2 h incubation with Srt^+^ (solid lines with increasing color intensity). For conversions based on peak integration, see Supporting Figure S6. Right: MS spectra for the selected peak (*) in the chromatogram including found m/z values (expected *m/z* signals for cy[2G‐α]: [M + 2H]^2+^ = 1181, [M + 3H]^3+^ = 788, [M + 4H]^4+^ = 591; cy[AIB‐2G‐α]: [M + 2H]^2+^ = 1107, [M + 3H]^3+^ = 738, [M + 4H]^4+^ = 554; cy[i4‐2G‐α]_2_: [M + 4H]^4+^ = 1169, [M + 5H]^5+^ = 936; cy[i7‐2G‐α]_2_: [M + 4H]^4+^ = 1196, [M + 5H]^5+^ = 957).

Following the CD‐based characterization of the helicity, we proceeded with peptide cyclization using a stabilized version of Sortase (Srt^+^). We chose this Sortase due to its advantages regarding handing and reproducibility. When performing kinetic measurements using 3G‐β as substrate (Supporting Figure S5), both Sortase versions (Srt* and Srt^+^) exhibit similar values for *K*_M_ (1720 vs. 1120 μM) and *v*_max_ (5.9 vs. 3.1 μM min^−1^). Reactions were performed at pH 7.5 using 100 μM of the respective peptide and 1 μM Srt^+^ (*T* = 37 °C) [22, 23]. In this case, we used higher Sortase concentration (*c* = 60 nM vs. 1 μM in the above described cyclizaions) to ensure high conversions. HPLC/MS analysis was performed before the addition of Srt^+^ (dashed line) and after 0.5, 1, and 2 h of incubation with the enzyme (Figure 4). For non‐modified 2G‐α, the analysis revealed efficient cyclization, providing already 65% of cyclic product cy[2G‐α] after only 0.5 h which further increased to 87% after 2 h (Figure 4A, left, Supporing Figure S6). The identity of cy[2G‐α] was confirmed by MS (Figure 4A, right). Similarly, the cyclization of AIB‐2G‐α proceeded efficiently, resulting in the formation of cyclic product cy[AIB‐2G‐α] (Figure 4B). The observed conversions aligned with the low helicity observed in the solution. The resulting structural flexibility presumably allows head‐to‐tail cyclization as observed above for the β‐sheet forming peptides (Figure 1).

When performing cyclization reactions with *i*,*i* + 4 stapled peptide i4‐2G‐α, we also observed the formation of a defined product, as indicated by the appearance of a new signal in the chromatogram (*, Figure 4C, left). However, the MS spectrum (Figure 4C, right) revealed the formation of a species with twice the molecular weight of the expected cyclic product cy[i4‐2G‐α] (expected *MW* = 2334 g mol^−1^, found *MW* = 4668 g mol^−1^). This indicates the formation of a cyclic product comprising two i4‐2G‐α sequences. The dimeric product, cy[i4‐2G‐α]_2_, can be explained by Srt^+^ performing the initial cleavage step but being unable to reach the N‐terminus of the same peptide. Instead, the transpeptidation occurs intermolecularly, forming a linear peptide dimer (Supporting Figure S7) which can subsequently act as a new substrate for Srt^+^. This longer sequence then exhibits sufficient flexibility to allow cyclization resulting in the cyclic dimer cy[i4‐2G‐α]_2_ (Supporting Figure S7). Interestingly, for most helical *i*,*i* + 7 stapled peptide i7‐2G‐α, incubation with Srt^+^ resulted in the appearance of a broad new peak in the chromatogram. This peak reveals a large number of unassigned MS signals and, again, though with lower abundance, a bicyclic species (cy[i7‐2G‐α]_2_, Figure 4D). Presumably, the increased helicity and rigidity of i7‐2G‐α, not only prevents monocyclization but also hinders the ability of the linear dimer to undergo head‐to‐tail cyclization. Taken together, we observe similar percent conversion for all four peptides after 0.5 h (54–65%, Supporting Figure S6), however, resulting in the formation of different products depending on the type of introduced peptide modification.

## 3 Conclusions

The secondary structure of peptides often plays a crucial role in their biological activity, as different structural motifs lead to distinct backbone arrangements [47, 48, 49, 50, 51]. For instance, in α‐helices, the N‐ and C‐termini are positioned on opposite sites of a relatively rigid rod, whereas in β‐hairpins, the termini are aligned in close proximity. In contrast, a random coil exhibits high flexibility, resulting in stochastic variations in the relative positioning of the termini. Building on these structural insights, we sought to explore how peptide α‐helicity influences Sortase‐mediated head‐to‐tail cyclization. To validate this approach, we first confirmed that an engineered Sortase efficiently cyclized a peptide with a β‐hairpin structure. Thereafter, we designed and synthesized peptides with varying helicity. Notably, both the non‐modified peptide and the peptide containing three AIB residues exhibited random coil characteristics and were readily cyclized by Sortase. This finding suggests that the inherent flexibility of these peptides allows the N‐terminus to attack the thioester within the peptide‐Sortase intermediate, thereby enabling intramolecular cyclization.

In contrast, increased α‐helicity in stapled peptide sequences inhibited monomer cyclization and instead yielded a cyclic dimer. This is likely due to an initial intermolecular reaction yielding a linear dimer which then exhibits sufficient flexibility to allow for cyclization. Such competition between inter‐ and intramolecular reactions has been reported previously for Sortase‐mediated reactions and, in our case, appears to be influenced by increased peptide rigidity [52]. Notably, both stapled peptides (*i*,*i* + 4 and *i*,*i* + 7) formed cyclic dimers. However, for the *i*,*i* + 7 stapled peptide, undefined additional products were observed suggesting that its increased rigidity inhibits monomer and to some extent also dimer cyclization thereby presumably triggering peptide oligomerization. Taken together, these findings highlight the critical role of secondary structure in shaping peptide physicochemical properties and function. More broadly, the observed correlation between peptide rigidity and enzymatic cyclization provides a notable example of how fine‐tuning secondary structures can direct the outcome of chemical reactions. These insights may contribute to the rational design of peptide‐based materials, hydrogels, and environment‐responsive probes in the future.

## Conflicts of Interest

The authors declare no conflicts of interest.

## Supporting information

Figure S1 β‐Sheet peptides: Analytical HPLC traces of purified peptides (3G‐β, 2G‐β, G‐β, β, 2G‐α, AIB‐2G‐α, i4‐2G‐α, and i7‐2G‐α), monitored at 210 nm. Right: Corresponding mass spectra displaying multiply charged species, including [M + 2H]^2+^, [M + 3H]^3+^, and [M + 4H]^4+^ ions, with their respective m/z values labeled. Each peptide exhibits characteristic mass peaks consistent with its expected molecular weight, confirming successful synthesis and purification (see Table S1).
Figure S2 HPLC‐MS analysis of cyclized peptide cy[3G‐β]. Left: HPLC traces showing the formation of cyclic peptide over time (0.5 h, 1 h, and 2 h) with the product peak marked by an asterisk (*). Right: MS spectrum of the cyclic peptide showing the characteristic [M + 2H]^2+^ and [M + 3H]^3+^ peaks at m/z 1083.3 and 722.9, respectively. Expected product m/z signals are cy[3G‐β]: [M + 2H]^2+^ = 1083.7, [M + 3H]^3+^ = 722.8.
Figure S3 Time course of β‐sheet peptide cyclization showing percent conversion over time for different peptide variants (β, G‐β, 2G‐β, and 3G‐β). The reaction was monitored for 210 min at RT, demonstrating increased conversion rates with additional glycine (G) residues (buffer: 20 mM HEPES, 150 mM NaCl, 5 mM CaCl2, 0.01% Tween‐20, 0.5 mM TCEP at pH = 7.5, peptide: *c* = 200 μM, Srt*: *c* = 60 nM).
Figure S4 α‐Helical peptides analyzed by HPLC‐MS. Left: Analytical HPLC traces of purified peptides (3G‐β, 2G‐β, G‐β, β, 2G‐α, AIB‐2G‐α, i4‐2G‐α, and i7‐2G‐α), monitored at 210 nm. Right: Corresponding mass spectra displaying multiply charged species, including [M + 2H]^2+^, [M + 3H]^3+^, and [M + 4H]^4+^ ions, with their respective m/z values labeled. Each peptide exhibits characteristic mass peaks consistent with its expected molecular weight, confirming successful synthesis and purification (see Table S1).
Figure S5 The Lineweaver–Burk plot of Srt* and Srt^+^ (*c* = 60 nM) using peptide 3G‐β as substrate (buffer: 20 mM HEPES, 150 mM NaCl, 5 mM CaCl₂, 0.01% Tween‐20, 0.5 mM TCEP, pH 7.5). Table provides kinetic parameters.
Figure S6 Conversion of linear pepjdes upon treatment with Srt^+^ based on integrajon of signals in analyjcal HPLC (for chromatograms see manuscript Figure 4, pepjde: *c* = 100 μM, Srt^+^: *c* = 1 μM, buffer: 20 mM HEPES, pH 7.5, 150 mM NaCl, 5 mM CaCl2, 0.01% Tween‐20, 0.5 mM TCEP).
Figure S7 Sortase‐mediated formajon of a cyclic pepjde dimer. Top: Schemajc representajon of the overall reacjon showing pepjde dimerizajon catalyzed by Sortase. Booom: Mechanism of the Sortase‐catalyzed transpepjdajon reacjon resuljng in a cyclic pepjde dimer. The enzyme recognizes the LPETG mojf and forms a thioester intermediate through its acjve site cysteine, followed by a nucleophilic aoack from the N‐terminal G of the second pepjde substrate, resuljng in the formajon of the linear dimer. Subsequent intramolecular aoack of the N‐terminus then provides the cyclic dimer. Gray boxes represent Sortase.
Table S1 Overview of pepjde sequences with amino acids related to Sortase‐mediated cyclizajon underlined. Observed and expected masses of 3G‐β, 2G‐β, G‐β, β, 2G‐α, AIB‐2G‐α, i4‐2G‐α, and i7‐2G‐α of the MS analysis (Figure S3) (*S*5: (S)‐2‐(4‐pentenyl)alanine, *R*8: (R)‐2‐(7‐octenyl)alanine), x: α‐ aminoisobutyric acid).

## Data Availability

The data that support the findings of this study are available in the supplementary material of this article.
