# Supplementary material for: Helicity‐Dependent Enzymatic Peptide Cyclization
Source: J Pept Sci. 2025 Apr 27;31(6):e70024. doi: 10.1002/psc.70024 (PMC12034914; doi:10.1002/psc.70024)
Supplement: Supplementary file 1 — Figure S1 β‐Sheet peptides: Analytical HPLC traces of purified peptides (3G‐β, 2G‐β, G‐β, β, 2G‐α, AIB‐2G‐α, i4‐2G‐α, and i7‐2G‐α), monitored at 210 nm. Right: Corresponding mass spectra displaying multiply charged species, including [M + 2H]2+, [M + 3H]3+, and [M + 4H]4+ ions, with their respective m/z values labeled. Each peptide exhibits characteristic mass peaks consistent with its expected molecular weight, confirming successful synthesis and purification (see Table S1). Figure S2 HPLC‐MS analysis of cyclized peptide cy[3G‐β]. Left: HPLC traces showing the formation of cyclic peptide over time (0.5 h, 1 h, and 2 h) with the product peak marked by an asterisk (*). Right: MS spectrum of the cyclic peptide showing the characteristic [M + 2H]2+ and [M + 3H]3+ peaks at m/z 1083.3 and 722.9, respectively. Expected product m/z signals are cy[3G‐β]: [M + 2H]2+ = 1083.7, [M + 3H]3+ = 722.8. Figure S3 Time course of β‐sheet peptide cyclization showing percent conversion over time for different peptide variants (β, G‐β, 2G‐β, and 3G‐β). The reaction was monitored for 210 min at RT, demonstrating increased conversion rates with additional glycine (G) residues (buffer: 20 mM HEPES, 150 mM NaCl, 5 mM CaCl2, 0.01% Tween‐20, 0.5 mM TCEP at pH = 7.5, peptide: c = 200 μM, Srt*: c = 60 nM). Figure S4 α‐Helical peptides analyzed by HPLC‐MS. Left: Analytical HPLC traces of purified peptides (3G‐β, 2G‐β, G‐β, β, 2G‐α, AIB‐2G‐α, i4‐2G‐α, and i7‐2G‐α), monitored at 210 nm. Right: Corresponding mass spectra displaying multiply charged species, including [M + 2H]2+, [M + 3H]3+, and [M + 4H]4+ ions, with their respective m/z values labeled. Each peptide exhibits characteristic mass peaks consistent with its expected molecular weight, confirming successful synthesis and purification (see Table S1). Figure S5 The Lineweaver–Burk plot of Srt* and Srt+ (c = 60 nM) using peptide 3G‐β as substrate (buffer: 20 mM HEPES, 150 mM NaCl, 5 mM CaCl₂, 0.01% Tween‐20, 0.5 mM TCEP, pH 7.5). Table provid [file PSC-31-e70024-s001.pdf]

Table of Contents

	page
Solid-Phase Peptide Synthesis	2
Peptide purification and Characterization	2
Circular Dichroism Assay	2
Peptide Cyclization	3
Supporting Figures	4
Supporting Tables	9
References	10

Solid-Phase Peptide Synthesis

Reagents were purchased from Iris Biotech GmbH, Sigma-Aldrich, Carl Roth, and Okeanos Biotech and used without further purification. Helical peptides were obtained from PSL Laboratories. β-Sheet peptides were synthesized via SPPS on H-Rink amide ChemMatrix® resin using an automated peptide synthesizer (Syro I, MultiSynTech GmbH, Witten, Germany), except for terminal glycine residues, which were manually coupled. The resin was swollen in DMF (dimethylformamide) for 30 min prior to usage. In manual synthesis a cycle of Fmoc deprotection, amino acid coupling, and capping was followed. The resin was washed with DMF (3×), DCM (3×), and DMF (3×) between reaction steps. Fmoc-removal was performed by suspending the resin in piperidine/DMF (2:8, v/v) (1 mL per 50 mg resin) twice for 5 min. Terminal glycine coupling was performed using Fmoc-Gly-OH (4 eq), COMU (4 eq), and Oxyma (4 eq) in DMF, activated with DIPEA (8 eq), and added to the resin (0.3 mL per 50 mg resin). The reaction was allowed to proceed for 20 min. A second coupling step was then performed using Fmoc-Gly-OH (4 eq), PyBOP (4 eq) in DMF (500 µL), followed by the addition of N-methylmorpholine (NMM) (8 eq). The crude peptide was obtained through simultaneous side-chain deprotection and cleavage from the resin using a mixture of TFA/H_2O/ODT/TIPS (94:2.5:2.5:1, v/v/v/v). The resin was treated twice with fresh portions of the cleavage cocktail, each for 1 h, and the filtrates were combined.

Peptide Purification and Characterization

Peptide purification was performed using reverse-phase high-performance liquid chromatography (RP-HPLC) on an Agilent 1100 semi-preparative system equipped with a Macherey-Nagel Nucleodur C18 column (10 × 125 mm, 110 Å, 5 µm). Separation was achieved with solvent A (H_2O + 0.1% TFA) and solvent B (ACN + 0.1% TFA) under optimized gradients over 20–60 min at a flow rate of 6 mL min^{-1}. Purified peptides were analyzed by analytical RP-HPLC coupled to electrospray ionization mass spectrometry (ESI-MS) using an Agilent 1260 system with a quadrupole 6120 detector. Chromatographic separation was performed on an Agilent Zorbax C18 column (4.6 × 150 mm, 5 µm) using solvent 5/95 (H_2O/ACN + 0.1% TFA) over 40 min. Peptides were quantified gravimetrically.

Circular Dichroism Assay

Peptides were prepared at a final concentration of 10 µM in an aqueous solution of 10 mM sodium phosphate (pH 7.4). Circular dichroism (CD) spectra were recorded at 20°C using a Jasco J-1500 spectropolarimeter and a 10 mm pathlength quartz cuvette (Hellma). Each spectrum was obtained from the average of three consecutive scans, acquired at a scanning speed of 100 nm·min^{-1} with a 1 mdeg sensitivity, 0.5 nm resolution, 1.0 nm bandwidth, and a 2 s integration time. Baseline correction was performed by subtracting the spectrum of the buffer alone, and the measured ellipticity (mdeg) was converted to mean residue ellipticity (MRE, deg cm^2 $dmol^{-1}$) using the formula below, normalizing for the number of peptide bonds (n) to facilitate direct comparison across peptide variants. Helicity values were calculated using the BestSEL[1-5] software tool (spectra range for calc.: 195-260 nm, developed by Micsonai et al.)

$$MRE\ (deg.cm^2.dmol^{-1}) = \frac{ellipticity\ (mdeg)\ x\ 10^6}{path\ length\ (mm)\ x\ protein\ concentration\ (\mu M) x\ n}$$

Peptide Cyclization

Sortase variants Str+ and Srt* were obtained as previously described.[6] Peptide cyclization was performed using Str+ (1 µM) or Str* (60 nM) in reaction buffer (20 mM HEPES, 150 mM NaCl, 5 mM $CaCl_2$, 0.01% Tween-20, 0.5 mM TCEP, pH 7.5). Peptides (*c* = 100–200 µM) were incubated at 37°C with agitation, and aliquots were taken at 0.5, 1, and 2 hours. Cyclization efficiency was monitored by HPLC-MS, assessing retention time shifts and mass-to-charge (m/z) ratios. For that purpose, an analytical RP-HPLC coupled to electrospray ionization mass spectrometry (ESI-MS) using an Agilent 1260 system with a quadrupole 6120 detector was used. Chromatographic separation was performed on an Agilent Zorbax C18 column (4.6 × 150 mm, 5 µm) using solvent 5/95 (H_2O/ACN + 0.1% TFA) over 40 min.

Supporting Figures

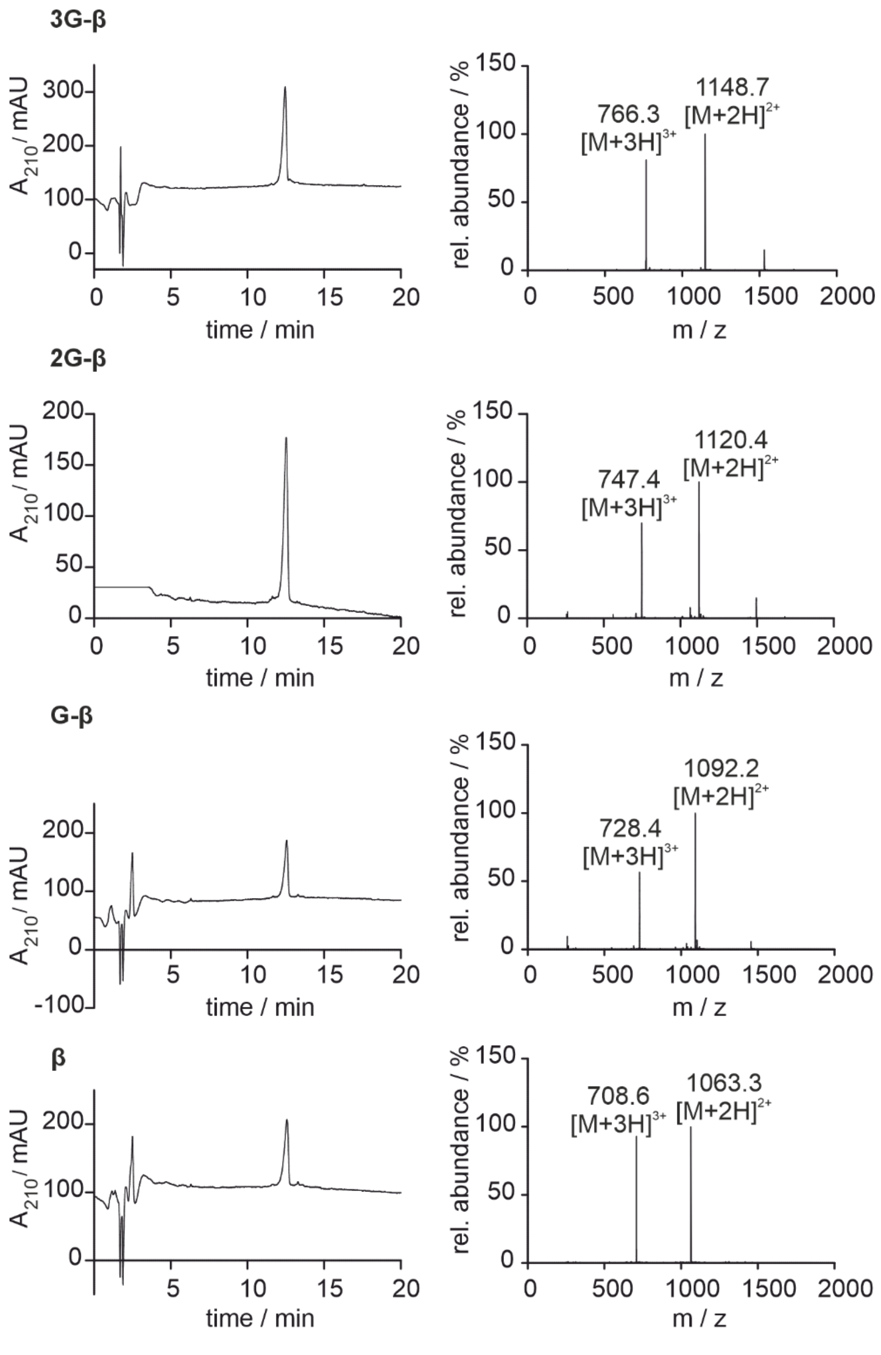

Figure S1│ β-Sheet peptides: Analytical HPLC traces of purified peptides (3G-β, 2G-β, G-β, β, 2G-α, AIB-2G-α, i4-2G-α, and i7-2G-α), monitored at 210 nm. Right: Corresponding mass spectra displaying multiply charged species, including $[M+2H]^{2+}$, $[M+3H]^{3+}$, and $[M+4H]^{4+}$ ions, with their respective m/z values labeled. Each peptide exhibits characteristic mass peaks consistent with its expected molecular weight, confirming successful synthesis and purification (see Table S1).

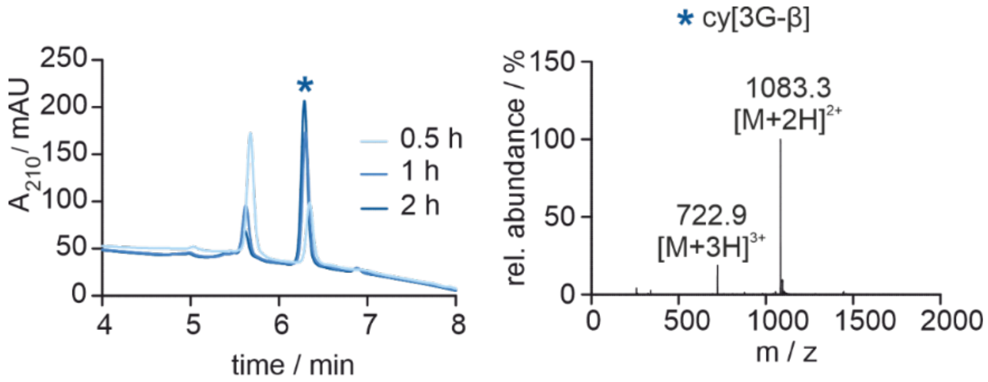

Figure S2│ HPLC-MS analysis of cyclized peptide cy[3G-β]. Left: HPLC traces showing the formation of cyclic peptide over time (0.5h, 1h, and 2h) with the product peak marked by an asterisk (*). Right: MS spectrum of the cyclic peptide showing the characteristic $[M+2H]^{2+}$ and $[M+3H]^{3+}$ peaks at m/z 1083.3 and 722.9, respectively. Expected product m/z signals are cy[3G-β]: $[M+2H]^{2+}$ = 1083.7, $[M+3H]^{3+}$ = 722.8.

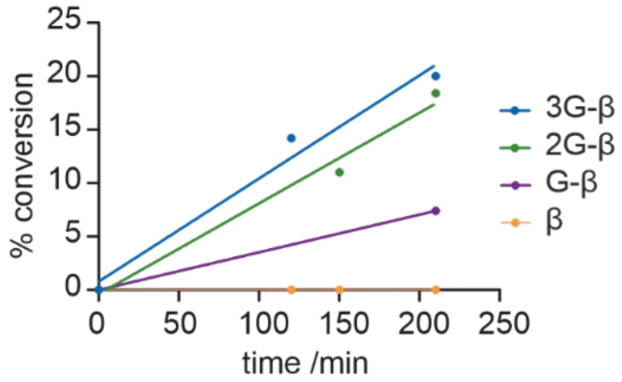

Figure S3| Time course of β-sheet peptide cyclization showing percent conversion over time for different peptide variants (β, G-β, 2G-β, and 3G-β). The reaction was monitored for 210 minutes at RT, demonstrating increased conversion rates with additional glycine (G) residues (buffer: 20 mM HEPES, 150 mM NaCl, 5 mM $CaCl_2$, 0.01% Tween-20, 0.5 mM TCEP at pH=7.5, peptide: c = 200 µM, Srt*: c = 60 nM).

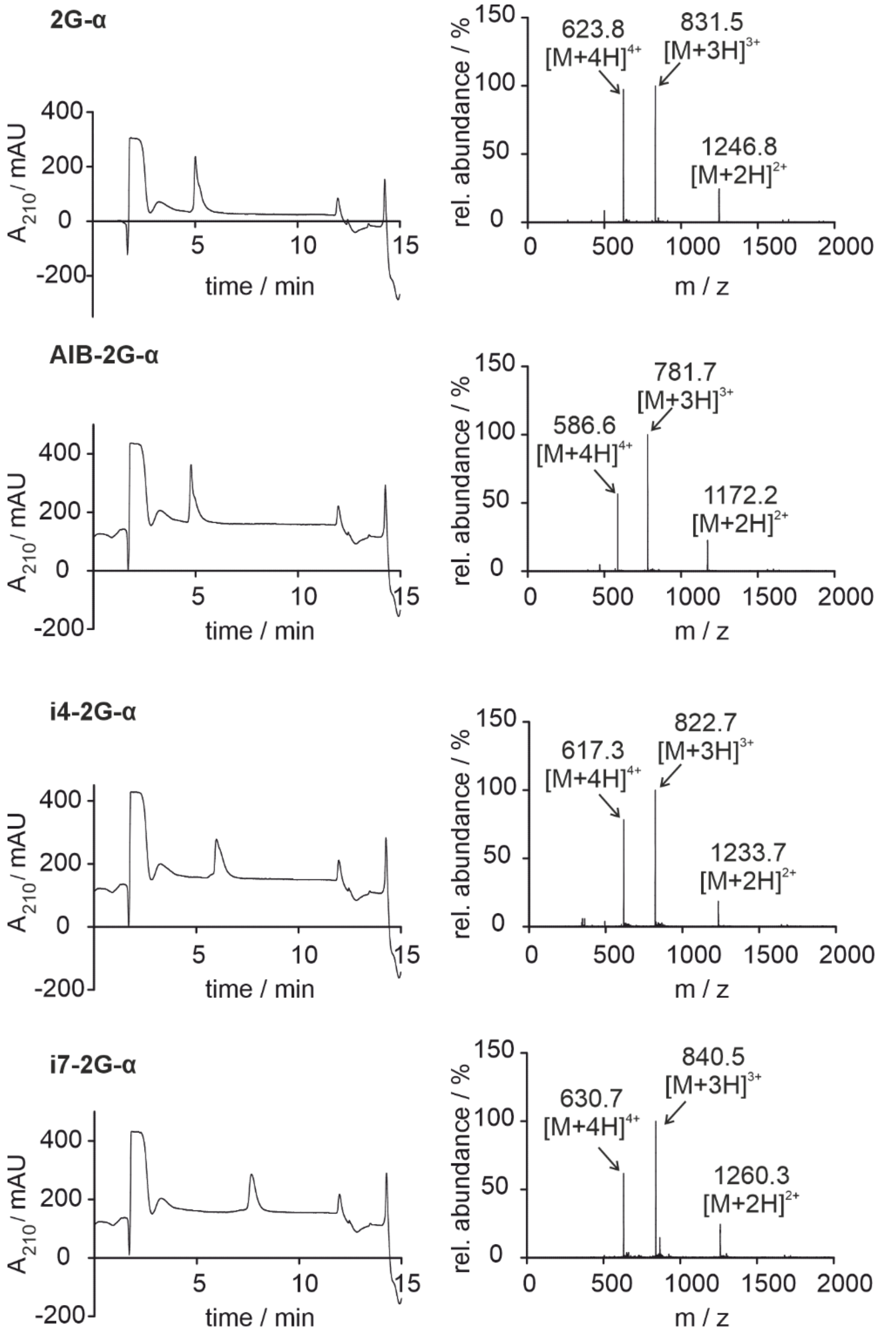

Figure S4│ α-Helical peptides analyzed by HPLC-MS. Left: Analytical HPLC traces of purified peptides (3G-β, 2G-β, G-β, β, 2G-α, AIB-2G-α, i4-2G-α, and i7-2G-α), monitored at 210 nm. Right: Corresponding mass spectra displaying multiply charged species, including $[M+2H]^{2+}$, $[M+3H]^{3+}$, and $[M+4H]^{4+}$ ions, with their respective m/z values labeled. Each peptide exhibits characteristic mass peaks consistent with its expected molecular weight, confirming successful synthesis and purification (see Table S1).

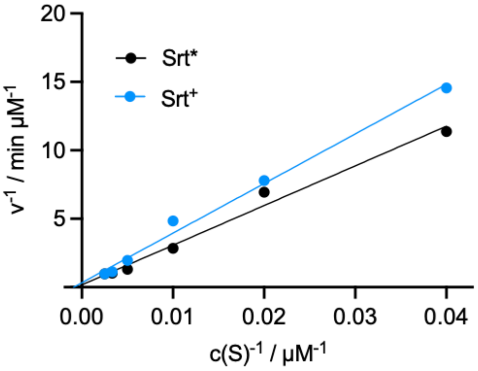

enzyme	K_M / µM	v_{max} / µM min^{-1}
Srt*	1720	5.9
Srt$^+$	1120	3.1

Figure S5| The Lineweaver-Burk plot of Srt* and Srt$^+$ (c = 60 nM) using peptide 3G-β as substrate (buffer: 20 mM HEPES, 150 mM NaCl, 5 mM $CaCl_2$, 0.01% Tween-20, 0.5 mM TCEP, pH 7.5). Table provides kinetic parameters.

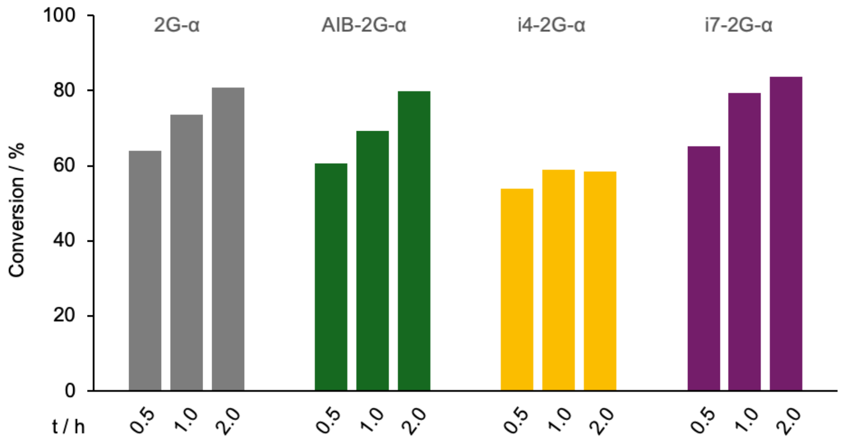

Figure S6│ Conversion of linear peptides upon treatment with Srt⁺ based on integration of signals in analytical HPLC (for chromatograms see manuscript Figure 4, peptide: c = 100 µM, Srt⁺: c = 1 µM, buffer: 20 mM HEPES, pH 7.5, 150 mM NaCl, 5 mM $CaCl_2$, 0.01% Tween-20, 0.5 mM TCEP).

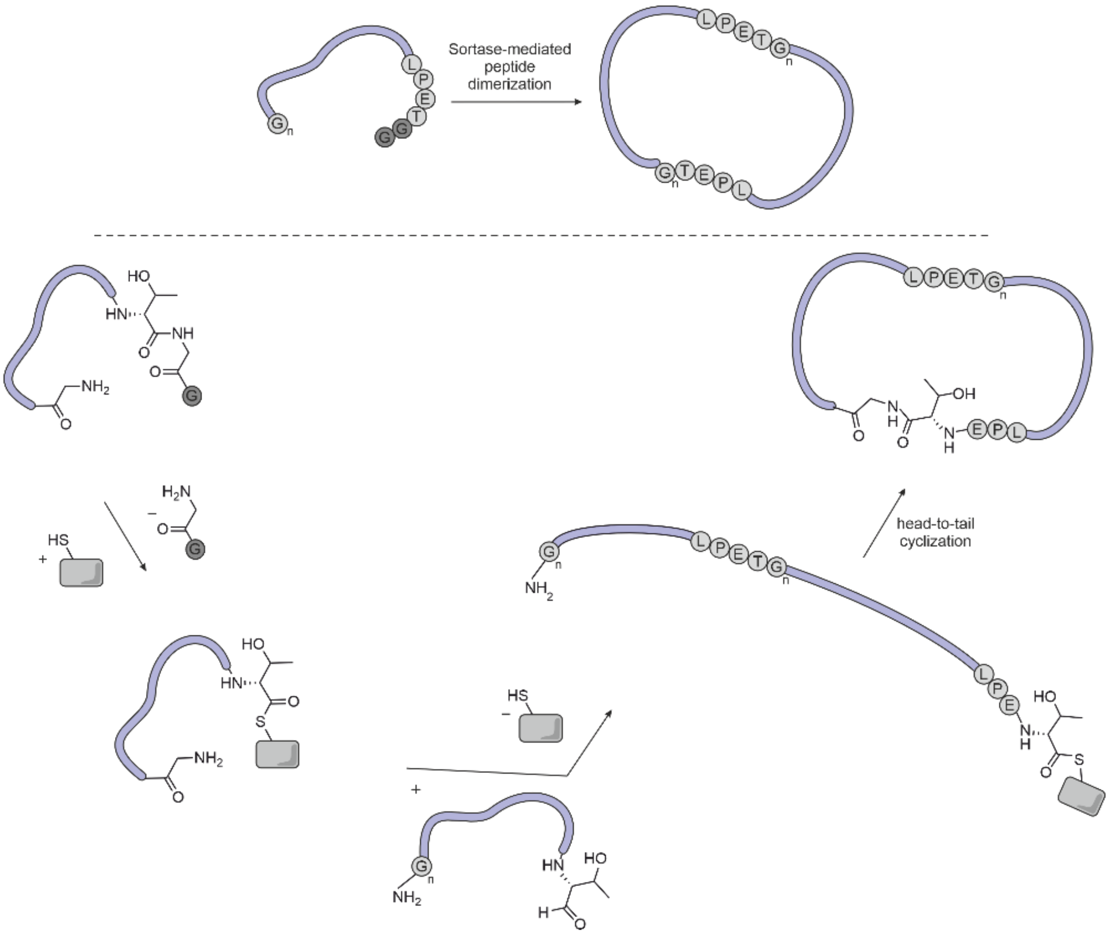

Figure S7│ Sortase-mediated formation of a cyclic peptide dimer. Top: Schematic representation of the overall reaction showing peptide dimerization catalyzed by Sortase. Bottom: Mechanism of the Sortase-catalyzed transpeptidation reaction resulting in a cyclic peptide dimer. The enzyme recognizes the LPETG motif and forms a thioester intermediate through its active site cysteine, followed by a nucleophilic attack from the N-terminal G of the second peptide substrate, resulting in the formation of the linear dimer. Subsequent intramolecular attack of the N-terminus then provides the cyclic dimer. Gray boxes represent Sortase.

Supporting Table

Table S1. Overview of peptide sequences with amino acids related to Sortase-mediated cyclization underlined. Observed and expected masses of 3G- β, 2G- β, G- β, β, 2G-α, AIB-2G-α, i4-2G-α, and i7-2G-α of the MS analysis (Figure S3) (S_5: (S)-2-(4-pentenyl)alanine, R_8: (R)-2-(7-octenyl)alanine), x: α-aminoisobutyric acid).

Peptide	Sequence	m/c calc.	m/z found
**3G-β**	GGGVTRNDVpPDSLLVFLPETGG	766.8 $[M+3H]^{3+}$<br>1149.7 $[M+2H]^{2+}$	766.3 $[M+3H]^{3+}$<br>1148.7 $[M+2H]^{2+}$
**2G-β**	GGVTRNDVpPDSLLVFLPETGG	747.8 $[M+3H]^{3+}$<br>1121.2 $[M+2H]^{2+}$	747.4 $[M+3H]^{3+}$<br>1120.4 $[M+2H]^{2+}$
**G-β**	GVTRNDVpPDSLLVFLPETGG	728.8 $[M+3H]^{3+}$<br>1092.7 $[M+2H]^{2+}$	728.4 $[M+3H]^{3+}$<br>1092.2 $[M+2H]^{2+}$
**β**	VTRNDVpPDSLLVFLPETGG	709.8 $[M+3H]^{3+}$<br>1064.2 $[M+2H]^{2+}$	708.6 $[M+3H]^{3+}$<br>1063.3 $[M+2H]^{2+}$
**2G-α**	GGQYHRILKRRQARAKLPETGG	623.9 $[M+4H]^{4+}$<br>831.6 $[M+3H]^{3+}$<br>1246.9 $[M+2H]^{2+}$	623.8 $[M+4H]^{4+}$<br>831.5 $[M+3H]^{3+}$<br>1246.8 $[M+2H]^{2+}$
**AIB-2G-α**	GGQ**x**HRI**x**KRR**x**ARAKLPETGG	586.6 $[M+4H]^{4+}$<br>781.9 $[M+3H]^{3+}$<br>1172.3 $[M+2H]^{2+}$	586.6 $[M+4H]^{4+}$<br>781.7 $[M+3H]^{3+}$<br>1172.2 $[M+2H]^{2+}$
**i4-2G-α**	GGQ$\boldsymbol{S}_5$HRI$\boldsymbol{S}_5$KRRQARAKLPETGG	617.4$[M+4H]^{4+}$<br>822.9 $[M+3H]^{3+}$<br>1233.9 $[M+2H]^{2+}$	617.3 $[M+4H]^{4+}$<br>822.7 $[M+3H]^{3+}$<br>1233.7 $[M+2H]^{2+}$
**i7-2G-α**	GGQY$\boldsymbol{R}_8$RILKRR$\boldsymbol{S}_5$ARAKLPETGG	630.7 $[M+4H]^{4+}$<br>840.6 $[M+3H]^{3+}$<br>1260.5 $[M+2H]^{2+}$	630.7 $[M+4H]^{4+}$<br>840.5 $[M+3H]^{3+}$<br>1260.3 $[M+2H]^{2+}$

References

1. Micsonai A, Wien F, Kernya L, Lee Y-H, Goto Y, Réfrégiers M, et al., Accurate secondary structure prediction and fold recognition for circular dichroism spectroscopy. *PNAS*. **2015**; 112(24):E3095-E103.

2. Micsonai A, Moussong E, Wien F, Boros E, Vadászi H, Murvai N, et al., BeStSel: webserver for secondary structure and fold prediction for protein CD spectroscopy. *Nucleic Acids Res*. **2022**; 50(W1):W90-W8.

3. Micsonai A, Moussong É, Murvai N, Tantos Á, Tőke O, Réfrégiers M, et al., Disordered–ordered protein binary classification by circular dichroism spectroscopy. *Front Mol Biosci*. **2022**; 9:863141.

4. Micsonai A, Wien F, Bulyáki É, Kun J, Moussong É, Lee Y-H, et al., BeStSel: a web server for accurate protein secondary structure prediction and fold recognition from the circular dichroism spectra. *Nucleic Acids Res*. **2018**; 46(W1):W315-W22.

5. Micsonai A, Bulyáki É, Kardos J. BeStSel: from secondary structure analysis to protein fold prediction by circular dichroism spectroscopy. New York, NY: Humana Press; 2021. 175-89 p.

6. Kiehstaller S, Hutchins GH, Amore A, Gerber A, Ibrahim M, Hennig S, et al., Bicyclic engineered sortase A performs transpeptidation under denaturing conditions. *Bioconjugate Chem*. **2023**; 34(6):1114-21.
